# The Foreign Journals

**Published:** 1837-01

**Authors:** 


### Art. XV.—THE FOREIGN JOURNALS. No. V.

### COLONIAL JOURNALS.

2.—*The Indian Journal of Medical and Physical Science. Edited by* Frederick Corbyn, Esq., Garrison Surgeon of Fort William.—*Calcutta*, 1836.

This is a monthly journal, published at Calcutta, and now in extensive circulation throughout the vast extent of our Indian possessions. It was established in January, 1834, by Messrs. J. Grant and J. T. Pearson, Surgeons, and edited by them until June, 1835; when its management was transferred to the present editor. It is got up in the same general style as our own weekly journals, with small print and double columns; but it is considerably larger, being of the royal octavo size, and consisting of at least two sheets and a half, or forty pages. Its price is sixteen rupees per annum, paid in advance, or two rupees the single number. Under the earlier management, this journal consisted for the most part of *Original Communications* from gentlemen resident in India, *Selections* from the British Journals, and *Medical Intelligence*, referring principally to India. The new series has somewhat extended the plan by giving Reviews of books (chiefly of Indian origin,) Hospital Reports, notices of the proceedings in the Calcutta Medical Society, Selections from the European Continental Journals, &c.

We have, we confess, been no less surprised than gratified by the perusal of this journal. We were by no means prepared for so much original matter and so much clever and spirited writing as it contains. It is extremely creditable to the editors, and speaks well for the zeal and intelligence of our medical brethren as a body, in our Indian empire; to whom this journal must be invaluable. We are much struck by the novelty and importance of many of the original communications on medical subjects, and we are only prevented from enriching our own pages with some of these by want of space. We cannot, however, resist the temptation of extracting a short passage from a series of very clever "Sketches of the Edinburgh Medical Professors," because it does just honour to one of the most enlightened and most estimable men in our profession. We hope it will not be ungratifying to the anonymous writer, nor yet to the subject of his well-merited eulogy, to see a portion of the picture reproduced in our pages:

"I claim the readers' company to the clinical wards of the Infirmary and then to the dark Lecture-room in the University, noted as the class room of old Dr. Andrew Duncan. In the first-mentioned place Dr. Alison is distinguished by the earnest mention he pays to the cases before him—by the pointedness and shrewdness of his professional questions, but, above all, by the kindness and urbanity of his manner to those whose chief claim upon him lies in the benevolence of his own excellent heart. The bland smile and the graciously put question receive in the chambers of the rich their mercenary, their pecuniary reward; but the reward which comes from the sick-bed of wretchedness and from the wards of indigence is, to a man of Dr. Alison's feeling, above all pecuniary valuation. Dr. Alison's treatment of the various diseases which the clinical wards presented, it would be tedious to notice—even if I could at this distant time bear in mind his system of treating every individual disease. It will be sufficient to mention, perhaps, that I think he was less bigoted to theories than his colleagues, treating disease more after symptoms than after names. In fever, this was particularly the case. And how strange it is to think that in a disease like fever, attacking all ages, sexes, temperaments—invading all organs and every texture, one line of practice should be laid down by every man as infallibly correct. It is this dogmatism in medicine that is the stronghold of quackery. Dr. Alison's clinical instruction was highly appreciated by the students. In diseases of the chest, one did well to watch his practice, above all, his diagnostic and prognostic inferences from the stethoscope. I think I have not known a physician whose practice showed more shrewd observative, or more profound professional knowledge. In the Clinical Lecture-room, Dr. Alison was, I think, unrivalled at the time I studied in Edinburgh. His remarks upon the particular case were so apposite; then, his application to it of his experience and of his theoretical knowledge was so convincing and so logical; and with all this flood of learning and practical instruction there was so little attempt at display—so little of mystery in the application of theory to practice—so much of sound sense, and of real utility, that you could not avoid feeling, that you had learnt something worth remembering, and that study and observation applied to the doctrines of medicine by the true logic—the induction from facts, must meet with its reward. A sketch of Dr. Alison cannot well be concluded without doing a little more than alluding to his philanthropy. He is a true Samaritan, pouring the oil into many a wound. To speak in plain English, there is not one man in the whole city that does more acts of charity: you will meet him in the very sinks of wretchedness, administering to the sick, expecting but one reward; but that, the greatest to him who can feel it—the approval of his own heart." (1835. No. xviii. p. 208.)

3. *The Jamaica Physical Journal.* Edited by William Arnold, m.d. —*Kingston, Jamaica,* 1836.

We know not the exact period of the origin of this Journal; the earliest Number which we have seen is dated September, 1834. It was then edited by James Paul, Esq. It will be seen by the title transcribed above that it has now a different editor; and it has also assumed a somewhat different aspect. It is published once in two months, in the usual octavo size, and contains six or seven sheets, or from ninety-six to

110 pages. We are sorry to say that we cannot compliment our brethren in Jamaica on the style in which the work is got up, as both paper and print are bad. The price of each Number is *ten shillings:* this seems high; but the British Colonies have always been remarkable for the depreciated value of money. Ten shillings in Jamaica currency (about 7*s*.) is probably not worth more than half-a-crown or three shillings in England.

We are happy to be able to speak in high terms of the contents of this Journal; these are, indeed, very creditable to the writers, and speak well for the state of medicine in the island. In the Numbers before us, coming down no further than April, 1836, we observe both medical and surgical papers, which indicate a great degree of science and operative skill. We regret that we cannot lay some of these before our readers in detail. The papers that please us most are those relating to the indigenous remedies and to the endemic diseases of the country and of the negro population. These are less numerous than we could wish; but some now published are very valuable. We trust that the editor's efforts will prove successful in stimulating the zeal of his brethren: if so, we are confident that we shall have to look to this publication for most important additions to our knowledge of the nature and treatment of tropical diseases. We know, by personal observation, that the diseases of the West Indies are very different from those of the East; and we hope that the practitioners of Jamaica will be able to throw as much light on the peculiarities of their endemic and epidemic maladies, as has been done, and as is still being done, by the practitioners of the Indian peninsula in regard to theirs.

From the notices in one of the earlier Numbers of this Journal, we glean that the Colonial Legislature granted a charter of incorporation, in 1833, to a "College of Physicians and Surgeons of Jamaica;" one object of which is to guard the medical profession and the public against the inroads of ignorant and unqualified practitioners. Dr. Bancroft, well known as the learned author of a very clever work on the Yellow Fever, very famous five and twenty years ago, was the president in 1834. We hope ere long to lay before our readers an authentic account of the State of Medicine and the Medical Institutions in this intelligent and very important colony.

### AMERICAN JOURNALS.

In proceeding with our notices of the American Journals, it is impossible, in looking at some of those now on our table, not to be struck with the pregnant proofs afforded by them of the marvellous rapidity and extent of the advance of civilization over regions which, even within our own days, were literally wildernesses, and, if inhabited at all, inhabited only by the rudest savages. If our noblest sympathies are engaged in contemplating the progress of human improvement and human enjoyment over the earth, our feelings of national pride are certainly not unconcerned when we see that in the South, in the East, and particularly in the West, Englishmen and the sons of Englishmen have been and are the main planners and promoters and actors in this great work. Only a few years ago, the very names of the cities where some of the Journals before us are published were hardly known in England; indeed, not very many years ago the cities themselves were not in existence: and when we consider how much must be done by a new community, how widely it must have spread, and how greatly it must have increased its numbers, before a *medical* Journal can be maintained, or could even be thought of, we need ask for no more convincing token of American aggrandisement in wealth and power and intellectual refinement, than the very existence of these publications. For ourselves, we must confess that we do not contemplate without emotion the springing up, year after year, on all the borders of the United States, of new institutions to instruct and benefit mankind, both physically and morally; and we hail every journal and book which we receive from our brethren of the far West as an affecting proof that our noble profession still keeps pace with the march of human amelioration.

5.—*The Western Journal of the Medical and Physical Sciences.* Edited by Daniel Drake, m.d., Professor of Medicine in Cincinnati College, and Wm. Wood, m.d.—*Cincinnati, Ohio,* 1836.

This Journal is published quarterly, each Number containing from 150 to 170 pages, and the four constitute one volume, price *three or four* dollars, according as the subscription is paid in advance or at the year's end. It has reached its thirty-sixth Number. It is well printed, and on good paper, and the whole publication is creditable to the editor. Like most of the other American Journals, it contains Original Communications, Reviews and Notices of Books, and Selections from the Journals of Europe and America. It gives a list of "collaborators," amounting to twenty, besides the editors; and most of them from the State of Ohio, or from States with names still less familiar to the English ear, as Indiana, Alabama, Illinois, Tennessee. Many of the articles, both in the Original and Review departments, are valuable, and we regret that the limits of our "Selections" department prevent us from extracting more largely from its pages.—Every now and then we observe in these Journals some little traits which indicate the simplicity and homeliness of the new community. For instance, in the April Number of this Journal, we observe appended to a list of the names of gentlemen who have paid their subscriptions the following note of the publisher's:

"One of the proprietors lost a letter, containing a remittance, a few days since, when he was leaving the post-office. He may yet find it; but, should he not do so, the money will be placed to the credit of the proper person, when further information is received. He remembers the residence of the individual, but has forgotten his name."

6. *The Transylvania Journal of Medicine and the associate Sciences.* Edited by L. P. Yandell, m.d., Professor of Chemistry in Transylvania University.—*Lexington, Kentucky,* 1836.

This is a quarterly Journal, on almost precisely the same plan as the Cincinnati Journal just noticed. It is, however, somewhat larger, both in the size and number of its pages; and the annual volume accordingly costs five instead of three dollars, (in advance.) It has existed nearly the same period as the other Journal, the Number for April, 1836, being the thirty-third. This Journal is announced as "emanating directly from the Transylvania School of Medicine," as being "identified with the profession in the West," and as "sustained by many of the ablest pens in the country;" and indeed it is but justice to say that it appears to us a very useful publication. The reviews in this, as well as in the Cincinnati Journal, are almost exclusively of works in English, either British or American; and we may remark, that we have observed the notice of works in the French or German language confined, in a great measure, to the Journals of the great Atlantic cities, which have a readier intercourse with foreign nations, and probably contain more writers capable of giving an account of works written in a foreign tongue.

We will thank our brother editors to continue to send us their Journals, in exchange for our own, through the medium of our agents in New York, Baltimore, and Philadelphia: but we must request them to remember that, in England, *books* cannot be sent by post, and that a small parcel sent by the packet across the Atlantic costs a large sum on its delivery in London.

### GERMAN JOURNALS.

11.—*Zeitschrift für die Staatsarzneikunde. Herausgegeben von* ADOLPH HENKE, Med. et Chir. Doct., Ordent. öffentl. Lehrer der Therapie, Klinik und Staatsarzneikunde, &c. &c., an der Königl. Bayerisischen Universität zu Erlangen, &c.—*Erlangen*, 1836.

*Journal of Forensic Medicine. Edited by* A. HENKE, M.D., Professor of Therapeutics, and of Clinical and Forensic Medicine in the University of Erlangen, &c.

THIS is a well-established and well-conducted journal, creditable at once to its learned editor and to the medical press of Germany. It has been established sixteen years, and has reached its sixty-third number and thirty-second volume. It is published at Erlangen, in quarterly numbers, each containing about fifteen sheets, or 240 pages, forming two volumes annually, of the price of three dollars twelve groschen, (about nine shillings English.) It is printed in the Roman type, but on the ancient whity-brown paper of Germany. We have no complaint to make of this Journal, but of its diffuseness.

The Forensic Journal of Henke consists entirely of original communications, and certainly contains many of great importance, in every department of forensic or state medicine, from the most eminent practitioners. It affords another instance of the high professional rank which is almost universally possessed by the editors of the German Journals. Besides holding the offices named in the title-page, and which we have only given in part, Dr. Henke is the author of numerous and valuable works in many departments of medical science, extending over no less a period than thirty years, viz. from 1806 to 1836.

In our present Number will be found a considerable extract from a recent Number of this work; and, as it must be generally admitted that the subject of Forensic Medicine is too little attended to in this country, we hope to be able, in every future Number, to transfer some portion of its valuable contents to our pages.

